# Usefulness of virtual reality-based training to diagnose strabismus

**DOI:** 10.1038/s41598-021-85265-8

**Published:** 2021-03-15

**Authors:** Hyun Sik Moon, Hyeon Jeong Yoon, Sang Woo Park, Chae Yeon Kim, Mu Seok Jeong, Sung Min Lim, Jee Heon Ryu, Hwan Heo

**Affiliations:** 1 Department of Ophthalmology, Chonnam National University Medical School and Hospital, 42 Jebongro, Dong-gu, Gwangju, 61469 Republic of Korea; 2 Department of Education, College of Education, Chonnam National University, Gwangju, Republic of Korea

**Keywords:** Ocular motility disorders, Eye manifestations

## Abstract

To study the usefulness of virtual reality (VR)-based training for diagnosing strabismus. Fourteen residents in ophthalmology performed at least 30 VR training sessions to diagnose esotropia and exotropia. Examinations of real patients with esotropia or exotropia before and after the VR training were video-recorded and presented to a strabismus expert to assess accuracy and performance scores for measuring the deviation angle and diagnosing strabismus with anonymization. A feedback survey regarding the usefulness and ease of use of the VR application was conducted for participants. The mean age of the 14 ophthalmology residents (10 men and 4 women), was 29.7 years. Before VR training, participants showed a mean accuracy score of 14.50 ± 5.45 and a performance score of 9.64 ± 4.67 for measuring the deviation angle and diagnosing strabismus in real patients with strabismus. After VR training, they showed a significantly improved accuracy score of 22.14 ± 4.37 (p = 0.012) and a performance score of 15.50 ± 1.99 (p = 0.011). According to the survey, most participants agreed on the usefulness of VR applications. This study suggests that VR-based training improved ophthalmology residents’ clinical diagnostic skills for strabismus in a short period.

## Introduction

Strabismus is not uncommon in children, and without appropriate approach and treatment, exacerbation of strabismus and subsequent sequelae, such as amblyopia, could occur. The ability to diagnose strabismus is an essential skill for ophthalmology specialists, and proper education and extensive clinical experience are required during residency training.

To date, methods such as practice-based learning, problem-based learning, team-based learning, and e-learning have been used to train medical specialists. In recent years, virtual reality (VR) and augmented reality (AR) simulation training have also been utilized, focusing on various medical fields. Advancements in VR represent some of the newest modalities being integrated into ophthalmologic practice and resident education^1–3^. The importance of incorporating simulation in the education of the residents and skills assessment is increasingly emphasized^4^. The 2019 coronavirus (COVID-19) pandemic also affected the medical curriculum; although junior doctors had to potentially serve as frontliners in this situation, they only had limited experience and education, both medically and surgically^5^. Therefore, the need for alternative educational strategies, such as VR simulation, has emerged^6,7^.

Therefore, we developed a VR application for diagnostic training of strabismus that could measure the deviation angle and diagnose strabismus using the head-mounted display (HMD) and VR technology. This study was aimed at investigating the usefulness of VR application as a training tool for ophthalmology trainees in the field of clinical strabismus.

## Methods

### VR application for diagnostic training of strabismus

The VR application was created based on the Oculus Rift (Oculus VR, LLC, Irvine, USA), the VR HMD, Oculus Touch (Oculus VR, LLC, Irvine, USA), wireless haptic controllers, and a computer running Windows 10 (Microsoft Corporation, Redmond, WA, USA).

The Oculus Rift HMD device was equipped with a liquid–crystal display (5.7″ diagonal, resolution of 1280 × 1440 pixels per eye), with a 110° field of view, mounted with an accelerometer, a gyroscope, and a magnetometer sensor for the positional tracking system. Oculus Touch controllers were peripheral accessories of the Oculus Rift and were employed to track the user’s hand position and orientation in a three-dimensional space. The HMD device and controllers were connected to a PC system.

**Figure 1:**
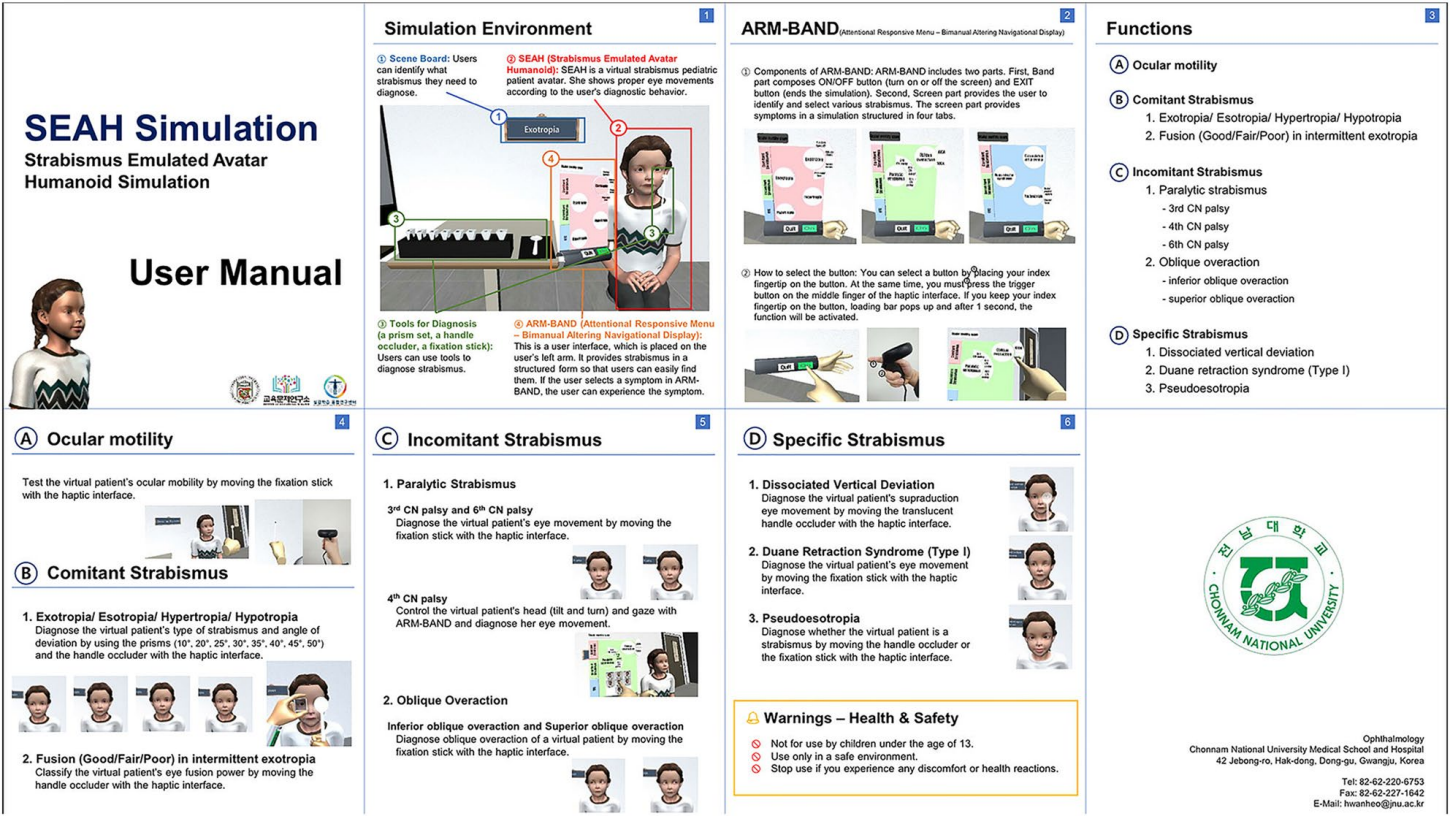
The user manual of the virtual reality application for diagnostic training of strabismus. The manual, which contains 8 pages, describes the simulation environment and the user interface implemented on the left wrist. The user interface provides lists of examinations that can be performed through simulation and how to carry them out.

A simple user manual was prepared to enable the usage of the VR application, and tutorials were also included in the application (Fig. 1). Users could use controllers to select and set the desired type of strabismus in the virtual environment. Thus, they could perform clinical tests and observe ocular motility in the various types of strabismus, such as horizontal, vertical, paralytic, and specific strabismus.

**Figure 2:**
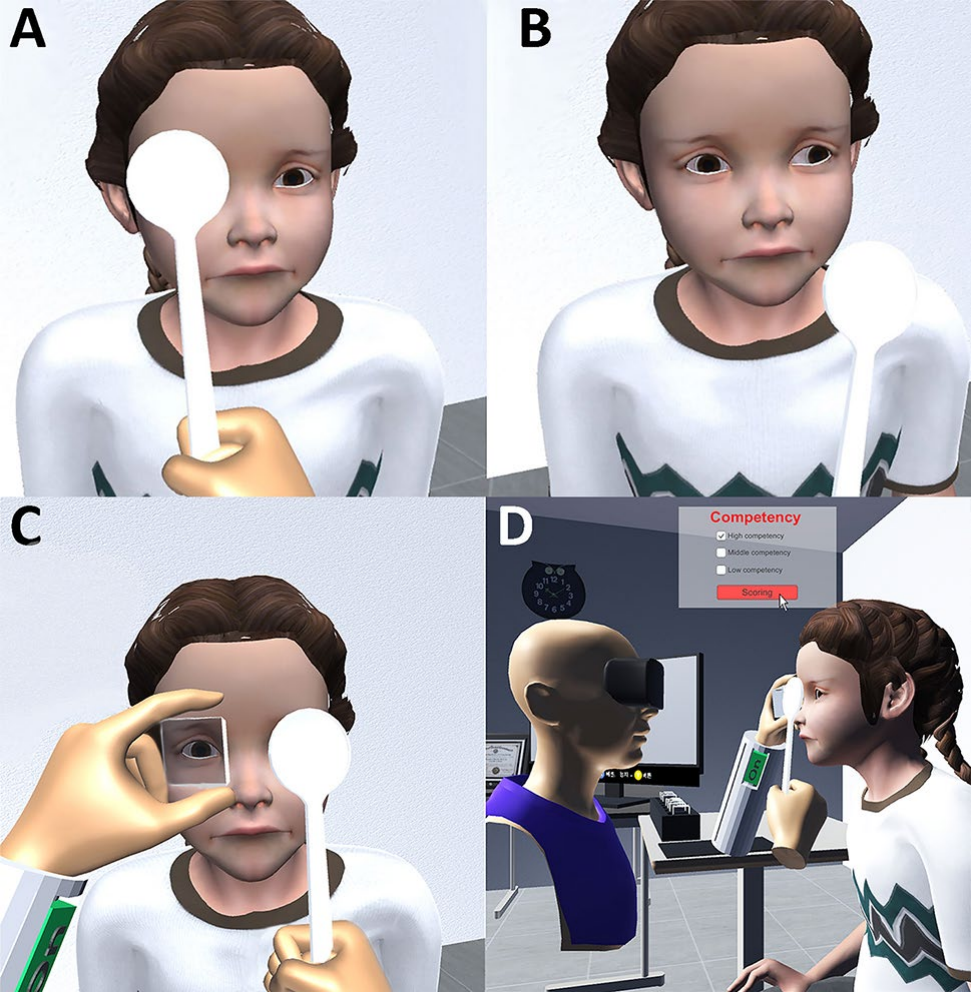
The virtual examination of virtual patients using an occluder and prisms implemented in virtual reality. **(A,B)** The cover-uncover test in a patient with left exotropia. **(C)** The prism cover test. **(D)** A user and a patient from the perspective of a third party.

In a VR environment, similar to an outpatient clinic, the strabismus patient was sited looking at a distant object, and the user was facing the patient from the right side. Furthermore, a virtual occluder and prisms with a spacing of five prism diopters were placed step by step on a virtually implemented table. The users could use the controllers to operate the virtual instrument. We also implemented cover-uncover, alternate cover, and prism cover tests to allow users to measure the patient's deviation angle (Fig. 2). In virtual patients with exotropia and esotropia, the non-dominant eye moves for fixation when the dominant eye is covered. In contrast, the dominant eye does not move when the non-dominant eye is covered. In the alternate cover test, an ocular movement is made, such that the type of strabismus could be distinguished. Furthermore, When the users perform the prism cover test with a smaller prism than the patient’s deviation angle, strabismus decreases but persists. In contrast, when a prism larger than the virtual patient’s deviation angle is applied, the eye moves in the opposite direction. When using a prism matching the patient’s deviation angle, no ocular movement is ascertained upon use (see Supplementary Video S1 online).

### Study design, setting, and participant

All participants provided written informed consent. The study complied with the tenets of the Declaration of Helsinki. The Institutional Review Board of the Chonnam National University Hospital approved the study protocol. This study followed the Standards for Quality Improvement Reporting Excellence Strengthening the Reporting (SQUIRE) guidelines for pre-post quality improvement study.

A total of 14 ophthalmology residents volunteered to participate in the study from the Chonnam national university hospital of South Korea between March 30, 2020, and July 31, 2020. Exclusion criteria were strabismus, previous ocular surgery, and ocular media's opacification, including cataract, and active ocular disease. The same person (H.S.M.) instructed participants with a brief standardized instruction on using this VR application, including a manual, tutorials of each module, and information about controlling the virtual occluder and prisms.

Participants carried out examinations on 3 actual patients with esotropia or exotropia, within 2 weeks before and after 30 sessions of VR training. The interval between the actual patient examination and before and after VR training was limited to two weeks. Each VR training session lasted at least 5 min, and participants performed at least one ocular motility examination, cover-uncover test, alternate cover test, and prism cover test on virtual patients. We implemented the application to count sessions wherein the completion requirement was met, and we recommended and confirmed them to run 30 sessions in 2 weeks. The virtual patient’s strabismus settings were limited to esotropia and exotropia between 20–40 prism diopter. We designed the study to ensure that each participant's pediatric ophthalmic training was not duplicated during the study period. They were given a little opportunity to see strabismus patients outside the pediatric ophthalmology clinic. We designed it such that each participants' pediatric ophthalmic training did not overlap with the study period.

### Evaluation

Examinations of actual patients were video-recorded and anonymous. After completing the process, videos were blinded as to which video recorded exams were conducted pre- versus post-VR training and presented to the strabismus specialist (H.H.) in a random order. The primary outcome measure was participants' accuracy and performance scores, measured by a predetermined scoring standard, which comprises specific procedures. To rate accuracy and performance, scores for 4 procedures, consisting of the ocular motility exam, cover-uncover test, alternate cover test, and prism cover test, were evaluated. Table 1 shows the accuracy score, with a total of 30 points, which was rated by scoring detail items for each procedure and diagnostic report. The performance score consisted of a total of 20 points, evaluated on a scale of 1 to 5 for mastery and natural performance for each item, where 1 means “poorly performed” and 5 means “performed well” (Table 2). Then, the scores of all participants before and after VR training were compared.

**Table 1:** Accuracy score rating standard of the diagnostic procedure for strabismus.

Procedures	Items to check	Score
Ocular motility examination	Using the instrument (1) Specifying the target (2) Checking the oblique muscles (2)	5
Cover–uncover test	Occlusion skill (5)	5
Alternate cover test	Occlusion skill (5)	5
Prism cover test	Prism-applying skill (3) Occlusion skill (2)	5
Diagnosis report	Accuracy of identifying the dominant and non-dominant eye/direction of deviation (3) Measured deviation angle (3) Strabismus diagnosis (4)	10
**Total score**		**30**

**Table 2:** Performance score rating standard of the diagnostic procedure for strabismus.

Procedures	Grade				
	Poorly performed	Performed with some errors or hesitation			Performed well
Ocular motility examination	1	2	3	4	5
Cover–uncover test	1	2	3	4	5
Alternate cover test	1	2	3	4	5
Prism cover test	1	2	3	4	5
**Total score**					**20**

The performance score was evaluated on a scale of 1 to 5, where 1 meant “Poorly performed” and 5 meant “Performed well”.

Subsequently, participants completed a feedback survey on the usefulness and ease of use of the VR application. Participants were asked to quantitatively rate their perceptions of the VR application on a scale of 1 to 5, where “1” meant “Disagree” and “5” meant “Agree” (Table 3)^8^.

**Table 3:** Results of the virtual reality application experience survey from participants.

Questions	Mean value
**Perceived usefulness**	
The application improves my understanding of the processes involved in the diagnosis of strabismus	4.43 ± 0.49
The application improves my strabismus inspection ability	3.86 ± 0.64
The application made it easier to observe the anomalies related to ocular position and movement	4.14 ± 0.35
The application will give me the confidence to perform this task on someone in the future	3.86 ± 0.64
**Average mean score**	4.07
**Perceived ease of use**	
Learning to use the application would be easy for me	3.57 ± 1.18
I find it easy to control virtual examination tools	3.71 ± 0.70
**Average mean score**	3.64

Participants' perception of virtual reality applications was quantitatively evaluated on a scale of 1 to 5, where 1 meant “Disagree” and 5 meant “Agree”.

Data were expressed as mean ± standard deviation unless otherwise indicated.

### Statistical analysis

Statistical analysis was performed using SPSS Statistics for Windows version 18.0 (IBM Corp., Armonk, NY, USA). Data are presented as mean ± standard deviation. The paired *t*-test was used to compare each participant's mean accuracy and performance scores before and after the VR application training. A p-value < 0.05 was considered to be statistically significant.

## Results

**Figure 3:**
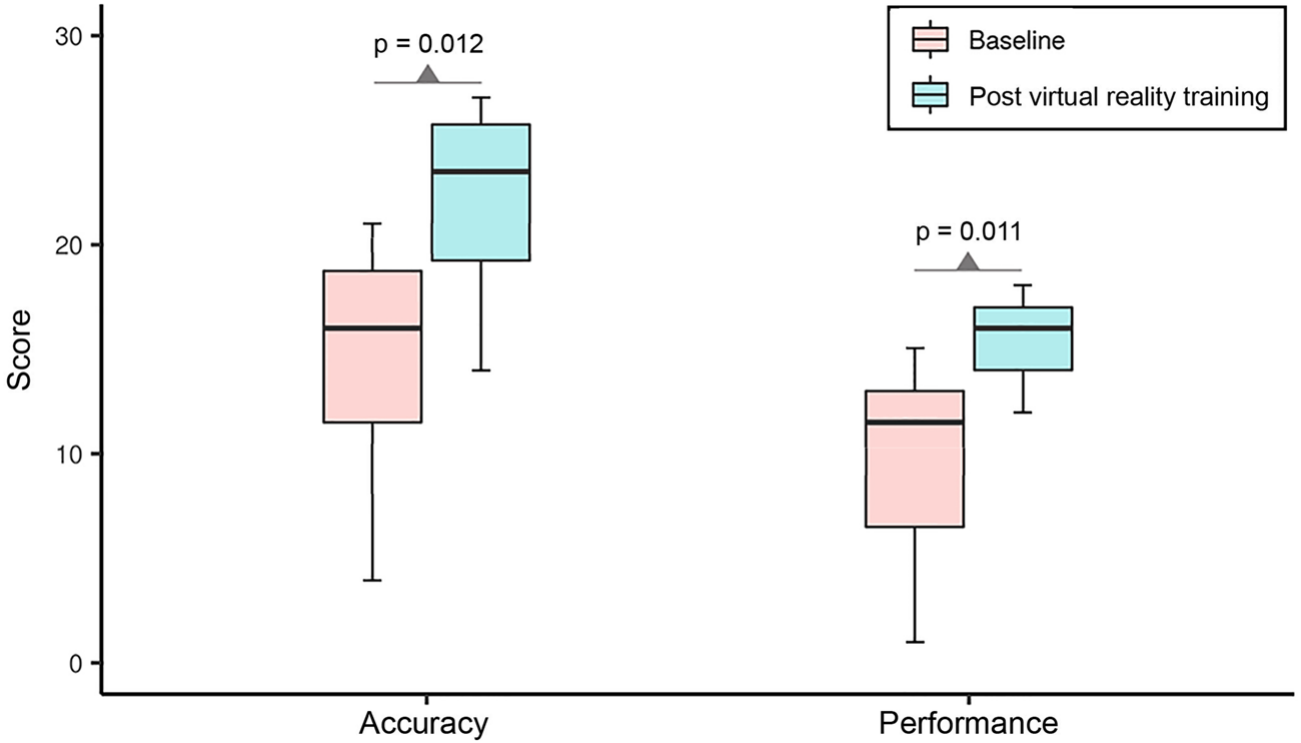
Comparisons of accuracy and performance scores before and after virtual reality training. After 30 sessions of virtual reality training for 2 weeks, participants’ accuracy and performance scores significantly improved (total accuracy and performance scores are 30 and 20 points, respectively; the paired *t*-test was used to compare changes in scores).

A total of 14 ophthalmology residents, including 10 men and 4 women, with a mean age of 29.7 years (range, 26–34 years), were enrolled in this study. No participants were excluded. The mean corrected visual acuity of participants was 0.00 LogMAR, and the mean stereoacuity with the Titmus Stereo test (Stereo Optical Co., Inc., Chicago, IL, USA) was 40 s of arc. When examining three real patients with strabismus before VR training, participants had a mean accuracy score of 14.50 ± 5.45 and a performance score of 9.64 ± 4.67. After 30 sessions of VR training for 2 weeks, participants again examined three real patients with strabismus and showed an accuracy score that had improved by 53% to 22.14 ± 4.37 (p = 0.012) and a performance score that had improved by 61% to 15.50 ± 1.99 (p = 0.011) (Fig. 3).

On a 5‑point rating scale, the overall score was 4.07 for the usefulness of the VR application and 3.64 for the ease of using the device and VR application (Table 3). Most participants agreed on the usefulness of the VR application, while some participants had a neutral opinion.

## Discussion

VR application for diagnostic training for strabismus could improve the accuracy and performance of residents’ skills to diagnose strabismus after performing only 30 sessions for 2 weeks. The analysis of the recording of participants’ examination on real patients with strabismus revealed that the participants’ skills of holding prisms, placing the occluder and performing cover-uncover and alternating occlusion improved after VR training. Participants were trained by applying a virtual occluder and prism to virtual patients, such as in the cover-uncover, alternate cover, and prism cover tests, which are essential tests to diagnose strabismus. Thus, users could determine both the type of strabismus and the angle of deviation. Although our VR application includes other vertical, incomitant, or specific strabismus. Our study was limited to participants performing VR training with virtual patients with exotropia and esotropia, which were implemented with various angles of deviation to assess the accuracy of the measured angle.

Studies regarding VR simulators and programs related to ophthalmic examination, treatment, and education have been reported. Tsapakis et al.^9^ developed a VR-based visual field (VF) test and reported that the results significantly correlated for glaucoma patients. Nakanishi et al.^10^ developed a VF test device by combining an electroencephalogram and electrooculogram with a VR device. As for the treatment, VR spectacles that could expand peripheral VF, HMD devices for low-vision rehabilitation, and vision enhancement were reported^11,12^. Besides, several studies on amblyopia and binocular vision using VR technology have also been conducted. A study about the improvement of visual acuity in patients with amblyopia through dichoptic training^2^, showed improvements in stereopsis in patients with intermittent exotropia^3^, and reported the assessment of ocular misalignment of strabismus patients through a dissociative test^13^. We have developed a system using HMD, combining the eye-tracking technology and VR, to measure the angle of strabismus with the principle of cover-uncover and alternate cover tests. We found that this system could identify ocular deviation with high accuracy and efficiency^14^.

Most reports on ophthalmology education using VR technology involved cataract surgery training using VR simulators. Thomsen et al.^1^ reported that clinically relevant cataract surgical skills could improve with proficiency-based training using a VR simulator, particularly for novices and intermediate-level surgeons. McCannel et al.^15^ presented that VR simulation could reduce the rate of errant capsulorhexis. Ng et al.^16^ reported that residents who had completed the VR training had greater confidence in performing phacoemulsification. In addition to surgical training, Wilson et al.^8^ reported that the VR ophthalmoscope training could successfully simulate performing eye examinations.

Residency training has not been easy or straightforward in the strabismus field, consistent with other fields. The process of accurately diagnosing strabismus is complicated and requires specialized skills.

During the current COVID-19 pandemic, the medical and surgical experience and education of residents were limited^5^. Recent research demonstrated that the demand for alternative technical educational tools to traditional medical and surgical education had increased, along with its use in practice: for web-based teaching, telementoring telemedicine, self-directed learning, and in-person clinical encounters, including VR simulators^6,7,17^.

In such situations, this VR application, as in other aforementioned educational programs, could play a role in allowing residents to improve their skills free from the burdens of space and time limitations. Additionally, residents developed the confidence to examine and diagnose children with strabismus. Although the VR application in this study was aimed to help ophthalmology residents, it could be beneficial for trained ophthalmologists to maintain or practice their skills.

There is a well-developed strabismus simulator to teach basic strabismus^18^. However, our VR program has more advantages than the simulator. Examiners can inspect using prisms and occluder by controlling both hands' haptics in the virtual clinic that was implemented like a real environment in a three-dimensional space. It makes the examiners feel the real appearance of the examination of strabismus patients. And the virtual patients' eye movements could be observed not only from the front but also from various directions, and the movement of the eyes covered by the occluder could be observed. Virtual patient’s eyes could accurately react to different virtual prism powers. These characteristics could be applied to objective structured clinical examination or clinical performance examination for the education of medical students. An additional advantage was being able to train on the VR program without a specific simulator, such as for cataract surgery training, and only requiring a VR headset and wireless haptic controllers. Thus, learners could train without time and space constraints.

A limitation of our study was the lack of a control group. It was not possible to conduct a randomized controlled trial because of the small sample size. It would have been optimal to randomize one group of residents to VR training while another group recieved no training or examined real patients with strabismus. The lack of a control group led to the risk of confounding factors and bias. To address this risk, we applied the generalizability theory to the before and after study design. The natural learning curve for regular education schedules for residents could affect the results as a confounding factor. To remove this element, we let them conduct VR training only for 2 weeks. We also restricted their rotation in the pediatric ophthalmology clinics or seeing strabismus patients during the study. Besides, there was one evaluator (H.H.) for rating participants’ examinations. This could lead to the risk of subjective assessment. To compensate for objectivity, we prepared the detailed rating scales in advance. Further, the recorded videos were also processed as much as possible so that the evaluator could not identify the participants. We provided the videos in random order to the evaluator so that it was not possible to check when they were recorded. Additional limitations were the small number of participants and the inclusion of horizontal strabismus alone. Including more participants and various types of strabismus would have enabled multiple regression analysis, with the experience level and complexity of strabismus as variables. Currently, the virtual patient model is being improved through a continuous update of the application and VR environment to determine various strabismus types in addition to the disease entity. Moreover, a real-time evaluation mode should be developed within the VR environment in order to create a proficiency-based education program beyond repetitive and time-based education. Based on these, we intended to evaluate the efficacy of training on the various types of strabismus and compare them among various groups and other strabismus training simulators in the future.

In conclusion, the results of our study suggest that the VR application for diagnostic training for strabismus could improve the accuracy and performance of examination skills of ophthalmology residents in a short term.

## Supplementary Information

Supplementary Legend.

Supplementary Video.

## Data Availability

The datasets generated during the current study are available from the corresponding author on reasonable request.
