# Supplementary material for: Usefulness of virtual reality-based training to diagnose strabismus
Source: Sci Rep. 2021 Mar 15;11:5891. doi: 10.1038/s41598-021-85265-8 (PMC7961051; doi:10.1038/s41598-021-85265-8)
Supplement: Supplementary file 1 — Supplementary Legend. [file 41598_2021_85265_MOESM1_ESM.docx]

**Supplementary Online Content**

**Usefulness of Virtual Reality-based Training to Diagnose Strabismus**

Hyun Sik Moon, M.D.^1^, Hyeon Jeong Yoon, M.D.^1^, Sang Woo Park, M.D., Ph.D.^1^, Chae Yeon Kim, B.A.^2^, Mu Seok Jeong, B.A.^2^, Sung Min Lim, B.A.^2^, Jee Heon Ryu, Ph.D.^2^, Hwan Heo, M.D., Ph.D. ^1^*

^1^Department of Ophthalmology, Chonnam National University Medical School and Hospital, Gwangju, Republic of Korea

^2^Department of Education, College of Education, Chonnam National University, Gwangju, Republic of Korea

**Supplementary Video S1.** Performing a strabismus examination with the virtual reality application.

In a VR environment, the users could measure the patient's deviation angle and diagnose the type of strabismus by conducting cover-uncover, alternate cover, and prism cover tests with the virtual instrument.
